# Hydroxyapatite bioactivated bacterial cellulose promotes osteoblast growth and the formation of bone nodules

**DOI:** 10.1186/2191-0855-2-61

**Published:** 2012-11-22

**Authors:** Neftaha Tazi, Ze Zhang, Younès Messaddeq, Luciana Almeida-Lopes, Lisinéia M Zanardi, Dennis Levinson, Mahmoud Rouabhia

**Affiliations:** 1 Groupe de Recherche en Écologie Buccale, Faculté de Médecine Dentaire, Université Laval, 2420, rue de la Terrasse, Québec, QC, G1V 0A6 Québec, Canada; 2 Département de chirurgie, Faculté de médecine, Université Laval, Centre de recherche de l'Hôpital Saint-François d'Assise, CHUQ, Québec, QC, Canada; 3 Département de physique, génie physique et d'optique, Faculté de Sciences et génie, Université Laval, Québec, QC, Canada; 4 DMC Equipamentos, Sao Carlos, SP, Brazil; 5 Department of medicine, University of Illinois, Chicago, USA

**Keywords:** *Acetobacter xylinum*, Hydroxyapatite, Osteoblasts, Tissue regeneration, Cellulose

## Abstract

The goal of this study was to investigate the feasibility of bacterial cellulose (BC) scaffold to support osteoblast growth and bone formation. BC was produced by culturing *Acetobacter xylinum* supplemented with hydroxyapatite (HA) to form BC membranes (without HA) and BC/HA membranes. Membranes were subjected to X-ray photoelectron spectroscopy (XPS) analysis to determine surface element composition. The membranes were further used to evaluate osteoblast growth, alkaline phosphatase activity and bone nodule formation. BC was free of calcium and phosphate. However, XPS analysis revealed the presence of both calcium (10%) and phosphate (10%) at the surface of the BC/HA membrane. Osteoblast culture showed that BC alone was non-toxic and could sustain osteoblast adhesion. Furthermore, osteoblast adhesion and growth were significantly (p ≤0.05) increased on BC/HA membranes as compared to BC alone. Both BC and BC/HA membranes improved osteoconductivity, as confirmed by the level of alkaline phosphatase (ALP) activity that increased from 2.5 mM with BC alone to 5.3 mM with BC/HA. BC/HA membranes also showed greater nodule formation and mineralization than the BC membrane alone. This was confirmed by Alizarin red staining (ARS) and energy dispersive X-ray spectroscopy (EDX). This work demonstrates that both BC and BC/HA may be useful in bone tissue engineering.

## Introduction

Developing effective bone regeneration therapy is a clinically important long-term goal. Bone loss caused by trauma, neoplasia, reconstructive surgery, congenital defects, or periodontal disease is a major health problem worldwide. Indeed, close to 6 million fractures occur annually in the United States. Of these, 5 to 10% (0.3 to 0.6 million) fail to heal properly due to non-union or delayed union (Franceschi 2005; Bostrom et al., 1999). In the case of periodontal disease, nearly half of adults between the ages of 45 and 65 have moderate to advanced periodontitis and associated alveolar bone loss, which, if not reversed, will result in the loss of almost 6.5 teeth/individual (Oliver et al. 1998). Clearly, there is a need for safe, effective methods to replace and promote bone regeneration. Advances in bone regeneration therapy will require innovative molecular biology and tissue engineering-based technologies. The regeneration of complex bone structures such as joints, craniofacial structures, or even entire bones and teeth will involve vastly more complex challenges including appropriate scaffold.

Scaffold is crucial in tissue engineering as a delivery vehicle for cell transplantation, a three-dimensional template for tissue regeneration, and as a matrix for signal transduction to regulate bone formation (Harris et al., 1998). Scaffold also serves as a reservoir for different molecules such as water and nutrients, and for various cell mediators such as cytokines and growth factors. Recent literature underscores the key role of the scaffold in influencing cell and tissue function (O'Brien et al., 2007; Bitar et al., 2008). Scaffold must be biocompatible to overcome adverse inflammatory responses and preferably be biodegradable to yield space for new tissue formation (Holland and Mikos 2006). The physical (porosity, topography) and chemical (material composition) properties of scaffold material should also suit a specific application. For example, to mimic native bone a scaffold must be osteoconductive and preferably, osteoinductive (Hasegawa et al., 2007). Type I collagen, hydroxyapatite and other calcium phosphate biomaterials are commonly used in bone tissue engineering (Wahl et al., 2007). These materials promote osteoblast and osteoprogenitor attachment, and differentiation to enhance bone formation (Wahl et al., 2007; Wang et al., 2007; Huang et al., 2007). Naturally derived materials such as collagen have limitations such as weak mechanical properties, while undesirable degradation profiles hamper the utility of hydroxyapatite. To overcome these limitations naturally derived materials can be combined with synthetic materials offering improved mechanical properties. Indeed, synthetic biodegradable polymers such as polyglycolide (PGA), polylactide (PLA), poly(*ε*-caprolactone) (PCL) and their copolymers have been widely used to fabricate clinically useful scaffolds (Gong et al., 2007; Buxton and Cobourne 2007). These were further improved by introducing natural proteins such as collagen, gelatin and growth factors (Garg et al., 2012). Many of the mechanical properties of these synthetic polymers are found in bacterial cellulose.

Bacterial cellulose (BC) produced from *Acetobacter xylinum* is a biocompatible polymer with excellent physical and chemical properties characterized by high tensile strength, elastic modulus and hydrophilicity (Helenius et al., 2006). Morphologically, the fibrous structure of BC is similar to the collagenous fibers of bone. These characteristics support BC as a useful scaffolding material in regenerative medicine (Bäckdahl et al., 2006; Andrade et al., 2010; Hong et al., 2006). Fang et al. 2009 recently reported a HA/BC nanocomposite scaffold supporting the growth and differentiation of human bone marrow stromal cells (MSCs). Also, recent studies have focused on the fabrication and characterisation of HA/BC as a composite material using a different method of appetite incorporation (Grande et al., 2009; Bodin et al., 2006) although, without consideration of the interaction of these composite materials with cell viability and function. However, study of BC as a bone regeneration scaffold is preliminary and the data are limited. The purpose of this study was to investigate whether BC and BC-hydroxyapatite (HA) can promote osteoblast growth and bone nodule formation.

## Materials and methods

### Production of BC membrane

*Acetobacter xylinum* (ATCC 52582) obtained from the American Type Culture Collection (Rockville, MD) was cultivated in 20 mL of medium in 100 mL flasks for 120 h at 28°C in static culture. The nutrient medium contained 2 wt% glucose, 0.5 wt% peptone, 0.5 wt% yeast extract, 0.27 wt% disodium hydrogen phosphate and 0.115 wt% citric acid. Bacterial cellulose pellicles were harvested and cleaned by immersion in 2 wt% NaOH solution at 80°C for 1 h. The pellicles were then immersed in 1 wt% NaClO solution for 30 min, washed with deionized water and sterilized by autoclaving (121°C for 15 min).

### Production of BC supplemented with HA (BC/HA)

Two methods were used to introduce HA into BC.

*Surface deposition of HA onto BC*. HA was formed in BC hydrogel by performing alternating incubation cycles with calcium and phosphate solutions (Hutchens et al., 2006). Briefly, BC membranes were incubated in a solution of CaCl_2_ (11 g/L) at pH 4.83 under agitation in an orbital shaker for 12 h at 23°C. The membranes were rinsed with deionized water and then incubated in a Na_2_HPO_4_ solution (8.52 g/L) for 12 h. The samples were then rinsed in deionized water and dried at 60°C to constant weight. The mass of HA in the cellulose was determined by subtracting the total composite weight from the average weight of control cellulose membranes. HA weight percentages were calculated by dividing the mass of the HA by the total composite weight. This was estimated at 50% of HA in the BC/HA membrane.

*Hydroxyapatite mixture with the bacterial cellulose pulp*. BC hydrogel was mechanically cut into slices to generate a BC pulp of 10% cellulose. After homogenization, BC solution was supplemented with hydroxyapatite at a 0.33% as previously reported (Varma and Babu 2005). The mixture was then vigorously stirred, poured into rectangular molds and dried at room temperature before use.

### X-ray photoelectron spectroscopy (XPS) analysis

The surface chemical elements of BC, BC/HA(50%) and BC/HA(0.3%) membranes were analyzed with a PerkinElmer PHI 5600 XPS (Eden Prairie, MN) with a standard magnesium X-ray source (1253.6 eV). The emitted photoelectrons were detected at a 45° take-off angle and analyzed with a hemispheric electron energy analyzer operated at a pass energy of 187.9 eV for the survey scans. For each membrane three locations of 0.8 × 0.8 mm^2^ each were analyzed and averaged. The vacuum in each sample chamber was maintained at 10–10 torr during measurement. All measurements were done at the air-facing side of the membranes, which is the side that faced air during membrane preparation.

### Osteoblast culture

Saos2 osteoblast-like cells, a human osteosarcoma cell line with osteoblastic properties, were used in this study. The osteoblasts were cultured in a 3:1 mixture of Dulbecco-Vogt’s modified Eagle’s (DME) medium and Ham’s F-12 (H) (Invitrogen Life Technologies, Burlington, ON, Canada) supplemented with 24.3 μg/ml adenine, 10 μg/ml human epidermal growth factor (Chiron Corp., Emeryville, CA, USA), 0.4 μg/ml hydrocortisone (Calbiochem, La Jolla, CA, USA), 5 μg/ml bovine insulin, 5 μg/ml human transferrin, 2 × 10^-9^ M 3,3’,5’-triiodo-L-thyronine, 100 U/ml penicillin, 25 μg/ml gentamicin (Schering, Pointe-Claire, QC, Canada), and 10% foetal calf serum (NCS, fetal clone II; Hyclone, Logan, UT, USA). Sub-confluent cell cultures were trypsinized; cells were split 1:10 to maintain cell growth, and were subsequently incubated at 37°C in a humid 8% CO_2_ atmosphere.

### Hoechst staining

Osteoblasts were seeded onto the different BC membranes at 5 × 10^5^ for two days. Adherent cells were subjected to Hoechst staining. The samples were first fixed with methanol/glacial acetic acid (75/25) for 3 × 15 min, and washed 3 times with PBS. They were then incubated with Hoechst 33342 (H42) (Riedel de Haen, Seele, Germany) (1 μg/ml) in PBS for 15 min at room temperature in a dark atmosphere. After three washes with deionized water, the samples were observed and photographed using an epifluorescence light microscope (Axiophot, Zeiss, Oberkochen, Germany).

### Lactate dehydrogenase assay

Since LDH is a soluble cytosolic enzyme that is released into the culture medium following loss of membrane integrity resulting from either apoptosis or necrosis (Melo et al., 2007), it was used in our experiments to assess possible cytotoxicity resulting from BC membranes. Osteoblasts were seeded onto BC and BC/HA membranes and then cultured for 2 days. At this time medium was refreshed and cells were cultured for an additional two and four days. Culture supernatant was collected at days two and four for LDH assessment; 4 and 6 day cultures respectively after seeding. LDH activity was measured using an LDH cytotoxicity assay (Promega, Madison, WI), per the manufacturer's protocol. Briefly, 50 μl of each supernatant were transferred to a 96-well flat-bottom plate and supplemented with 50 μl reconstituted substrate mix. The plate was incubated in the dark at room temperature for 30 min. This assay is based on the conversion of L-lactate and NAD to pyruvate and NADH by released LDH (Gleitz et al., 1996). To stop the reaction a volume of 50 μl of an acid solution was added to each well, after which approximately 100 μl of each reacted solution was transferred to a 96-well flat-bottom plate, and absorbance read at 490 nm with a X-Mark Microplate Spectrophotometer (Bio-Rad, Mississauga, ON, Canada). A positive control for total LDH activity release was added to the experiment. Both positive (PC) and negative controls (NC) were included. LDH release was calculated using the following formula:

$$\begin{array}{ll} \text{Total LDH release}\left(\%\right)= & \left(({\text{BC}}_{\text{absorbance}}-{\text{NC}}_{\text{absorbance}}\right)\\ & ÷({\text{PC}}_{\text{absorbance}}-{\text{NC}}_{\text{absorbance}}))\\ & \times 100\% \end{array}$$

### MTT assay of osteoblasts cultured on BC membranes

Osteoblasts (5 × 10^5^) were cultured on BC/HA membranes for 2, 4 and 6 days before being subjected to MTT [(3-(4,5-dimethylthiazol-2-yl)-2,5-diphenyltetrazolium-bromide)] assay as we previously reported (Denizot and Lang 1986). BC membranes that do not contain hydroxyapatite were used as control. A second control, i.e., BC/HA and BC membranes that were not seeded with osteoblasts, was also included. This allows for the measurement of non-specific adsorption of MTT to the BC membranes. Osteoblasts on each membrane were cultured in the presence of 1% (v/v) MTT solution (5 mg/mL) for 4 h, after which the supernatant was removed and the cultures were washed with PBS. Then 1 mL of HCl in isopropanol (0.04*N*) was added and incubated for 15 min. At the end of the incubation, 200 μL (in triplicate) of solution was transferred from each membrane to a 96-well flat-bottom plate, and the absorbance of the MTT (formazan) was determined at 550 nm using an ELISA reader (Model 680, BioRad Laboratories, Mississauga, ON, Canada).

### Alkaline phosphatase assay

Alkaline phosphatase (ALP) activity in the supernatant of osteoblast cultured membranes was measured. In preparation for this assay, Saos2 cells (5 × 10^5^) were seeded on BC, BC/HA(50%) and BC/HA(0.3%) membranes and allowed to adhere overnight. Medium was refreshed and cells were cultured for 2 and 4 days at 37°C in a 5% Co_2_ humid atmosphere. At the end of each culture period, medium was collected and centrifuged twice at 3000 rpm for 10 min to eliminate cell debris and ALP enzyme activity was assayed. One hundred μL of collected culture medium was supplemented with 100 μL of substrate solution (100 mg of 4-nitrophenyl phosphate disodium salt hexahydrate in 25 mL H_2_O) and 20 μL of alkaline buffer solution (1.5 M 2-amino-2-2 methyl-1-propanolol at pH of 10.3) in a 96 well plate, and the plate was incubated for 1 hour at 37°C. The reaction was stopped by adding 100 μL 0.3 M NaOH to each well. Optical density was read at 405 nm using an X-Mark microplate spectrophotometer (Bio-Rad, Mississauga, ON, Canada) and translated into ALP enzyme activity using a standard curve generated with p-nitrophenol (Sigma-Aldrich) ranging in concentration from 0 to 20 mM.

### Qualitative and quantitative analyses of nodule formation

Osteoblasts (5 × 10^4^) were cultured on BC and BC/HA membranes for 3 and 4 weeks. Membranes were washed twice with PBS and then stained with Alizarin Red S (ARS) solution for 2 min before being washed three times with sodium acetate buffer solution (pH 6.3) as we have previously reported (Meng et al., 2011). Mineral nodules were documented by photomicrography at random locations on each membrane. To quantitatively assay nodule mineralization, the ARS stain was dissolved with cetyl-pyridinium chloride (CPC) (Fisher Scientific, Ottawa, ON, Canada) for 1 h under gentle agitation. One hundred microliters of the CPC from each membrane were transferred to a 96-well plate and diluted with 100 μL of water. Absorbance of ARS was determined at 570 nm by means of the X-Mark Microplate Spectrophotometer (Bio-Rad, n = 4).

### Scanning electron microscopy (SEM) analysis

Osteoblasts (5 × 10^5^/cm^2^) were cultured on BC and BC/HA membranes for 2, 4 and 6 days. The membranes were rinsed three times with PBS, fixed in 4% paraformaldehyde for 15 min, and rinsed again four times in distilled water. Dehydration was performed in a series of ethanol solutions of increasing concentrations (50, 70, 90, and twice at 100%), with a 5-min dehydration treatment in each solution. The dehydrated specimens were kept overnight in a vacuum oven at 25°C, after which time they were sputter-coated with gold and examined with a JEOL 6360 LV SEM (Soquelec) operating at a 30 kV accelerating voltage. The experiment was repeated four times and representative photographs were taken (n = 4).

### Energy dispersive X-ray spectroscope (EDX) analysis

Osteoblasts (5 × 10^5^/cm^2^) were cultured on BC and BC/HA membranes for 2, 4 and 6 days. The specimens were then subjected to EDX analyses. The specimens were fixed with ethylene glycol for 30 min and dried at 50°C. Following sputter coating with Au-Pd, the specimens were analyzed by means of EDX using a JEOL 840-A SEM (JEOL, Tokyo, Japan).

### Statistical analysis

Experimental values are presented as means ± SD. The statistical significance of differences between the values was evaluated using a one-way ANOVA. *Posteriori* comparisons were done using Tukey’s method. Normality and variance assumptions were verified using the Shapiro-Wilk test and the Brown and Forsythe test, respectively. All of the assumptions were fulfilled. Data were analyzed using the SAS version 8.2 statistical package (SAS Institute Inc., Cary, NC, USA). Results were considered significant at < 0.05.

## Results

### Production of HA enriched bacterial cellulose membrane

**Figure 1:**
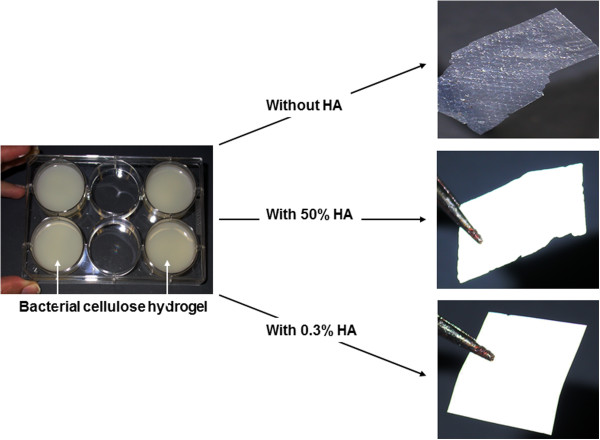
**Macroscopic observation of the different BC materials.** Note the difference in color and transparency between BC alone and BC supplemented with HA.

As shown in Figure 1, BC hydrogel appears as a 3-dimensional thick membrane. The membrane was then enriched with HA through alternating incubation cycles in CaCl_2_ followed by Na_2_HPO_4_ solutions at 25°C. After drying at 50°C, we observed a thin, opaque white BC membrane (Figure 1). The second process incorporating HA into BC pulp also gave a thin, opaque white BC membrane (Figure 1). In both HA enriched BC membranes there was 50% and 0.3% HA, respectively. To confirm the presence of HA in the BC membrane, we performed XPS analyses of the surface elemental composition (%) of the BC specimens. All measurements were done at the air-interface. As shown in Table 1, BC membrane alone has 57% carbon, 42% oxygen with no calcium and phosphate. In the BC membrane enriched with 50% of HA, there was 18% carbon, 49% oxygen, 10% calcium and 10% phosphate on the membrane surface. The level of calcium and phosphate was lower in the cellulose pulp-prepared BC membrane enriched with only 0.3% HA; 53% carbon, 46% oxygen, 0.4% calcium and 0.2% phosphate on the membrane surface. Overall, both methods led to the production of BC membranes with HA exposed on the surface, which was expected to be useful for osteoblasts growth.

**Table 1:** XPS surface element analyses

**Sample**	**Elements (%)**					
	**Carbon (C)**	**Oxygen (O)**	**Calcium (Ca)**	**Phosphate (P)**	**Chloride (Cl)**	**Sodium (Na)**
BC	57	42.6	0	0	0.2	0
BC-50%HA	18	49.5	9.9	9.7	2.1	10.5
BC-0.3%HA	53.4	45.9	0.4	0.2	0	0

BC: Bacterial cellulose, HA: Hydroxyapatite, XPS: X-ray photoelectron spectroscopy.

### BC with and without HA promoted osteoblast adhesion

**Figure 2:**
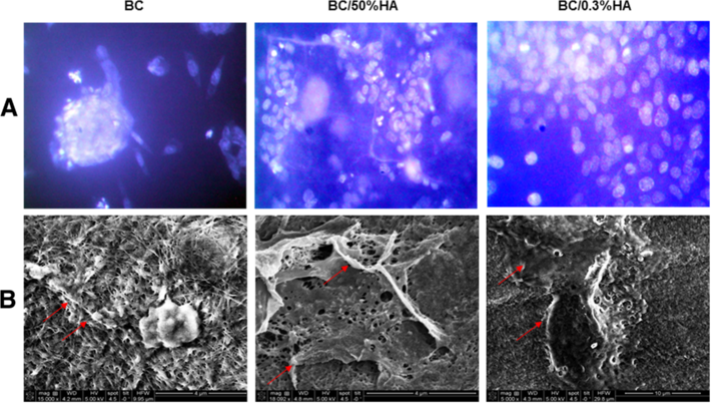
**Interaction between the osteoblasts and the different BC polymers.** Osteoblasts were cultured on the BC polymers for two days. (**A**) Adherent cells were subjected to Hoechst staining (*bar* 10 μm, n = 4). (**B**) SEM analysis of the BC polymers containing osteoblasts cultured for 2 days, showing cell adhesion (arrows).

**Figure 3:**
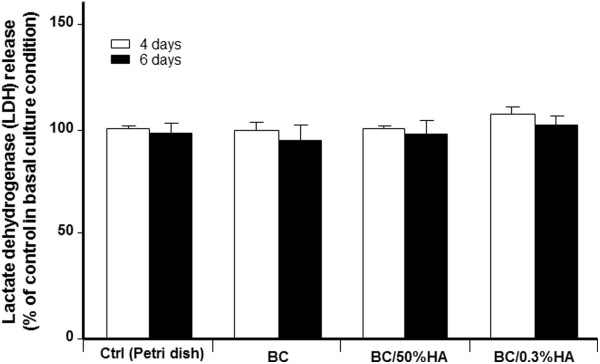
**BC polymer has no cytotoxic effect of osteoblasts.** Following osteoblast culture onto each BC polymer for 4 and 6 days, cultured supernatants were used to assess the LDH activity. Note the same levels of LDH with all tested conditions. Data are means + SD, *n* = 5.

Following osteoblast seeding and culture on BC membranes, cells were stained with Hoechst. As shown in Figure 2A, BC membranes supported cell adhesion and had no adverse effect on cell morphology. However, osteoblast adhesion was greater on the HA enriched BC membranes. This was confirmed by SEM analyses showing superior cell growth and spreading throughout the surface of the HA enriched BC membranes (Figure 2B). It is interesting to note that HA as low as 0.3% improved osteoblast adhesion. These results clearly demonstrate the compatibility of the BC membranes used in this study with osteoblasts. We have also shown that the addition of HA enhanced osteoblast adhesion and spreading. To further confirm the non-toxicity of BC, as shown in Figure 3, the release of LDH activity was comparable between BC membranes and tissue culture plates.

### BC with and without HA promote osteoblast proliferation

**Figure 4:**
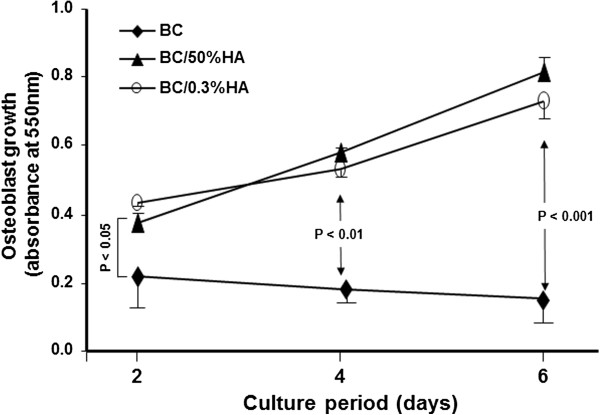
**Osteoblasts grow onto BC polymers.** Osteoblasts were seeded onto BC polymers and cultured for different periods. Cell growth was then assessed by mean of MTT assay, (*n* = 5). The difference was obtained by comparing the cell growth with BC alone to hydroxyapatite rich BC polymers. Difference was considered significant at *p* < .05.

Because osteoblasts adhered to the BC membranes, we assessed their proliferation potential using the MTT assay. As shown in Figure 4, BC was able to support osteoblast adhesion and growth. However, both HA enriched BC membranes demonstrated significantly higher osteoblast growth when compared to the BC membrane without HA. Osteoblasts cultured on the BC/HA membranes showed exponential growth up to 6 days. Interestingly, even at a level as low as 0.3%, HA promoted osteoblast proliferation which was compatible to osteoblast growth on BC membranes containing 50% HA. The overall results suggest that HA enriched BC is superior to BC alone for osteoblast proliferation. It should be noted that the lower osteoblast growth of BC membrane alone was not due to cellular toxicity of BC.

### Osteoblast cultured onto different BC materials secreted ALP

**Figure 5:**
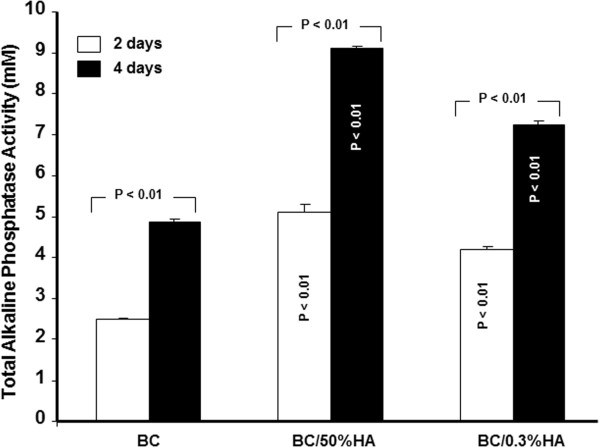
**BC polymer with and without hydroxyapatite promotes ALP activity.** Following culture onto BC polymers ALP activity was measured by alkaline phosphatase substrate p-NPP. Different levels of ALP activity were observed in the range of 5 to 9 mM with the hydroxyapatite rich BC polymers; compared with the BC polymer without hydroxyapatite. Data were presented as the mean + S.D. (*n* = 4).

Both BC and BC/HA membranes promote ALP production from osteoblasts (Figure 5), which increased with time of culture. For the BC membrane alone, ALP concentration went from 2.5 mM on day two to almost 5 mM on day four. The presence of HA in the BC membrane significantly promoted the ALP activity as compared to BC alone. Indeed, with the BC/HA(0.3%) membrane, ALP activity was more than 4 mM at day two and 7 mM at day four (p both < 0.01). Even greater ALP activity was seen with the BC/HA (50%) membrane; 5 mM at day two and 9 mM at day four. These data show that osteoblasts cultured on BC have ALP activity and furthermore, this activity increases when the BC polymer is supplemented with HA.

### BC promotes osteoblast mineralization

**Figure 6:**
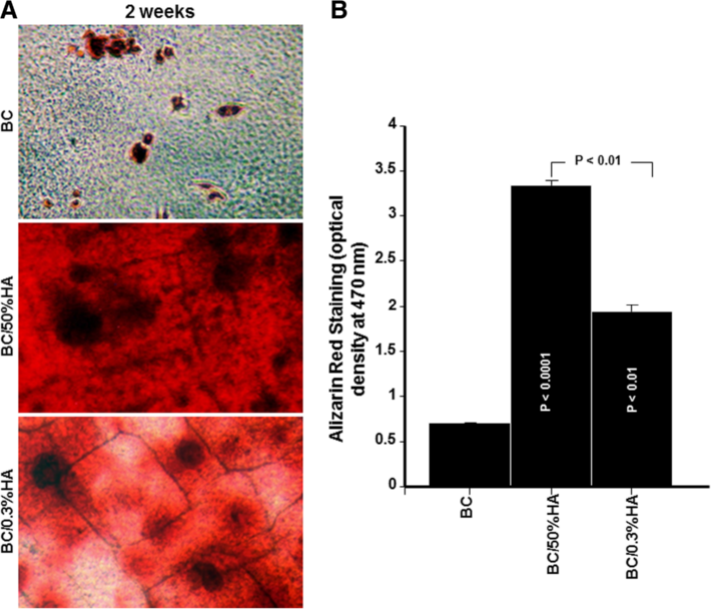
**BC polymer promotes Osteoblast mineralization.** Osteoblasts were culture onto the BC polymers for 2 weeks. Cells were fixed and stained with 0.5% alizarin red. Subsequently the alizarin red stain was eluted with cetylpyridinium chloride and measured quantitatively. Panel (**A**) photographs show the extent of mineralization (bar = 10 μm). Panel (**B**) graph represents the mean + SD (n = 5).

**Figure 7:**
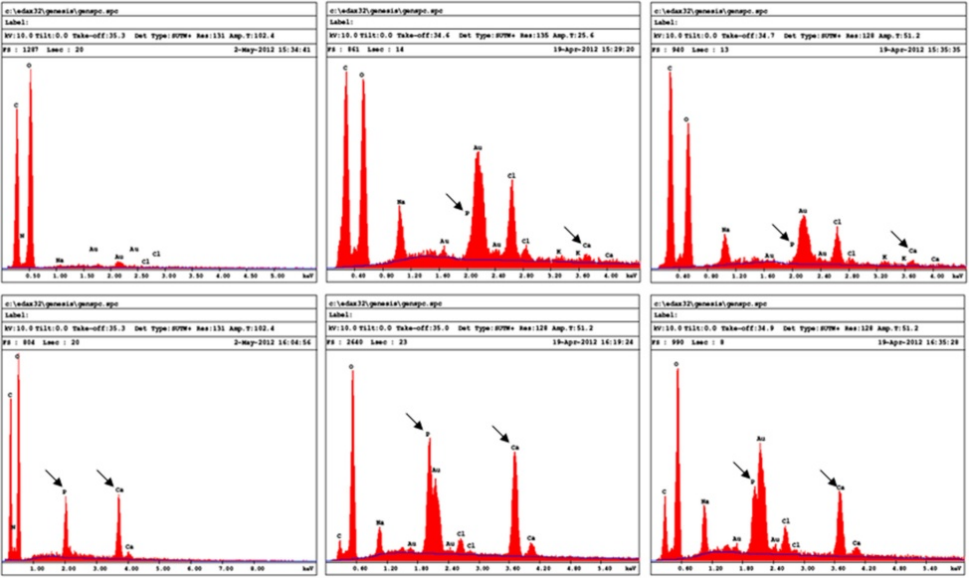
**EDX microanalysis of nodules osteoblast seeded and non-seeded BC polymers.** BC Polymers were seeded or not with osteoblasts then cultured for 6 days. They were then subjected to EDX analyses for phosphate and calcium element detection along with other elements. Element values were reported in Table 2. Representative grafts (n = 4).

Because osteoblasts were able to adhere to BC membranes and proliferate, we investigated the mineralization process using the ARS assay. As shown in Figure 6A, multiple nodules were observed after two weeks of culture. However, the number and size of nodules were greater for the HA enriched BC membranes than for the HA free BC membranes. This was confirmed by quantitative analyses using a CPC assay. ARS levels were significantly greater with the BC/HA membranes than with the BC membrane (Figure 6B). These data suggest the osteogenic potential of BC polymer which is synergistic with HA. Confirmation of mineralization was performed by EDX analysis. As shown in Figure 7, all of the EDX analyses revealed rich calcium and phosphate deposits on the surface of the osteoblast-seeded BC membranes as compared to the cell free membranes. The peaks referring to phosphate and calcium were greater in the osteoblast seeded BC polymers as compared to non-seeded membranes. This was supported by quantitative analyses shown in Table 2. Indeed, in HA free BC polymer the phosphate and calcium elements were undetectable. However, when osteoblasts were cultured for 6 days, the phosphate and calcium levels were 8 and 21 wt% respectively, demonstrating phosphate and calcium production by osteoblasts and deposition onto the BC polymer surface. The levels of both phosphate and calcium were further increased with HA enriched BC membranes seeded with osteoblasts (Table 2). Together, these data demonstrate the osteogenic nature of the BC polymer, and the possibility to improve this osteogenic property by adding a natural component to the BC membrane such as HA.

**Table 2:** Quantification of the surface chemical elements of the different BC polymers by EDX

**EDAX ZAF Quantification (Standardless)**						
**Element normalized**						
**SKC Table: Default**						
**Without osteoblasts**				**With osteoblasts**		
	**Element**	**Wt %**	**At %**	**Element**	**Wt %**	**At %**
BC	C K	44.28	53.71	C K	30.31	42.98
	N K	2.87	2.99	N K	6.38	7.76
	O K	46.73	42.55	O K	33.36	35.52
	P K	0.00	0.00	P K	8.07	4.44
	Na K	0.35	0.22	Na K	0.00	0.00
	Au K	5.48	0.04	Au K	0.00	0.00
	Cl K	0.29	0.003	Cl K	0.00	0.00
	Ca K	0.00	0.00	Ca K	21.88	9.30
	Total	100%	100%			
	**Element**	**Wt %**	**At %**	**Element**	**Wt %**	**At %**
BC-50%HA	C K	38.58	64.07	C K	4.67	11.76
	O K	19.15	23.89	O K	25.01	46.87
	Na K	3.00	2.60	Na K	2.18	2.84
	P K	1.13	0.73	P K	11.65	11.28
	Au K	27.29	2.76	Au K	25.45	3.87
	Cl K	8.55	4.81	Cl K	2.55	2.16
	K K	0.73	0.37	K K	0.00	0.00
	Ca K	1.54	0.78	Ca K	28.49	21.31
	Total	100%	100%			
	**Element**	**Wt %**	**At %**	**Element**	**Wt %**	**At %**
BC-0.3%HA	C K	46.09	68.54	C K	13.87	32.64
	O K	20.47	22.85	O K	21.50	37.99
	Na K	2.60	2.02	Na K	3.36	4.13
	P K	0.67	0.39	P K	6.09	5.56
	Au K	21.44	1.95	Au K	34.92	5.01
	Cl K	6.24	3.14	Cl K	4.02	3.20
	K K	1.03	0.47	K K	0.00	0.00
	Ca K	1.45	0.65	Ca K	16.25	11.46
	Total	100%	100%	Total	100%	100%

BC: bacterial cellulose, EDX: Energy dispersive X-ray.

## Discussion

In the present study we demonstrate the feasibility of producing bacterial cellulose and incorporating into this polymer, a native bone material, HA. HA incorporation was done through two processes using either BC hydrogel or BC pulp before polymer casting. This confirms previously reported data related to HA incorporation of bacterial cellulose (Hutchens et al., 2006; Wan et al., 2009). The presence of HA in the BC was confirmed by XPS analyses showing that even with low initial (0.3%) HA concentration, we were able to produce a calcium and phosphate rich BC membrane. The presence of BC surface calcium and phosphate is expected to promote cell adhesion and growth (Saska et al. 2011; Akkouch et al., 2011). This hypothesis is supported by osteoblast culture on different BC membranes showing that osteoblasts adhere to the surface of the polymer. However, the adherent cells were not able to sustain growth over time. In fact, proliferation decreases with time of culture. This is contradictory to what has been reported previously (Ferreira et al., 2010; Andersson et al., 2010; Saska et al., 2012). This difference could be due to the experimental condition used including seeding concentration. Indeed, Ferreira et al. ( 2009) showed that fibroblasts seeded at high concentration achieved better adhesion rates than low seeding concentration. Alternatively, a recent study reported low cell growth and low cell activity when cells were cultured on BC alone in comparison with a growth factor, BMP2 supplemented BC (Sun et al., 2012). It is important to note that the low adhesion and proliferation levels of osteoblasts on BC were not simply due to a cytotoxic effect. Indeed, the LDH level was not significantly different using BC as compared to BC enriched with HA. The potential reduction of cell number over culture time could be due an apoptotic process of differentiated cells (Shi et al., 2012), or simply due to the emergence of osteoblasts into a non-dividing G0 phase (Boissinot et al., 2012). Further work is required to address these issues.

The low adhesion and proliferation levels of osteoblasts were significantly (p<0.05) improved by the addition of HA (Blomquist et al., 1979). Indeed, as demonstrated in this study, adhesion and growth of osteoblasts onto HA supplemented BC were greater as compared to BC alone. For example, proliferation was 2, 3 and 6 times higher for the HA enriched BC than for HA free BC. The availability of HA at the surface improved both cell attachment and proliferation (Blomquist et al., 1979; Lock et al., 2012). Interestingly, even with low level (0.3%) HA in the BC membrane, osteoblasts adhere well and proliferate. The osteogenic activity of BC polymer was supported by increased ALP activity over culture time. However, ALP activity was greater in HA enriched BC than in BC polymer alone. This confirms a previously reported observation when BC was supplemented with another growth factor, BMP2 (Sun et al., 2012).

The bacterial cellulose environment promoting osteoblast adhesion and growth is favored by the presence of calcium and phosphate elements on the surface of the BC/HA (Polini et al., 2011; Strobel et al., 2012). In this environment, osteoblasts were able to form bone nodules. This is due to an active mineralization process through the secretion and deposition of mineralizing elements including calcium and phosphate on the surface of the BC membrane (Figure 7, Table 2). Indeed, following osteoblast culture on BC polymers, the levels of calcium and phosphate increased. In the HA free BC polymer, the calcium levels increased from zero with non-cell seeded BC to 21.8% in the osteoblast seeded BC polymer. The same observation was made with phosphate. This supports the hypothesis that osteoblasts maybe differentiating more than proliferating when cultured on BC polymer (Polini et al., 2011; Strobel et al., 2012; Ogata et al., 2012). Such differentiation would support the bone nodule formation we have shown. In conclusion, BC cellulose alone supports osteoblast adhesion but preserves the osteoblast in a non-dividing (G0) state. In this situation, osteoblasts differentiate leading to tissue mineralization through nodule formation as demonstrated by the high percentage of calcium and phosphate on the BC polymer surface. Interestingly, when BC is supplemented with HA, osteoblasts adhere, proliferate and mineralize better as compared to the BC polymer alone. Together, these data suggest that BC could be an appropriate support for bone tissue engineering. With the incorporation of active molecules such as HA and BMP2 the osteogenic potential of bacterial cellulose polymers may be optimized for multiple tissue engineering tools.

## Competing interest

The authors declare that they have no competing interests.
